# Mast cell burden and immunophenotype of Chinese patients with cutaneous mastocytosis: a 10-year study with focus on the easily neglected pathogenic features

**DOI:** 10.3389/fmed.2026.1828727

**Published:** 2026-06-22

**Authors:** Qiannan Jia, Xinran Li, Aiping Fan, Dian Zheng, Shuaixin Li, Weiqi Wang, Siying Li, Jun Li

**Affiliations:** Department of Dermatology, State Key Laboratory of Complex Severe and Rare Diseases, Peking Union Medical College Hospital, Chinese Academy of Medical Sciences and Peking Union Medical College, Beijing, China

**Keywords:** cutaneous mastocytosis, immunophenotype, maculopapular cutaneous mastocytosis, mastocytosis, pathological feature, telangiectasia macularis eruptive perstans

## Abstract

**Background:**

Cutaneous mastocytosis (CM) is a heterogeneous disease. Challenges remain in the detailed pathological features and pathogenic factors and their potential correlation in Chinese patients.

**Objective:**

This study aimed to characterize the pathological features and immunophenotypes of different CM subtypes in a real-world cohort and to explore the potential correlations between mast cell-related pathogenic features and clinical phenotypes.

**Methods:**

A cohort of 88 patients diagnosed with CM by histopathological examination in the Department of Dermatology between 2015 and 2024 was established. Immunohistochemical and clinicopathological analyses were performed.

**Results:**

Mast cell burden is associated with CM subtype, with the lowest burden being in telangiectasia macularis eruptive perstans (TMEP), followed by urticaria pigmentosa (UP) and diffuse CM/mastocytoma. High mast cell burden may also be associated with specific clinical morphology (e.g., elevated lesions) and an earlier age of onset. Different clinical subtypes exhibit distinct immunophenotypes. The expression of CD2, CD25, and CD30 may correlate with specific subtypes with high mast cell burden and provide potential value for CM diagnosis and subtyping. TMEP showed pathological differences from other subtypes, particularly in mast cell burden and immunophenotype.

**Conclusion:**

Different CM subtypes display distinct mast cell burdens and immunophenotypes, suggesting potential diagnostic value. The diverse clinical phenotypes may be associated with different mast cell burdens. TMEP may represent a unique and rare morphological variant of UP.

## Introduction

Mastocytosis represents a heterogeneous group of hematologic disorders characterized by abnormal proliferation and accumulation of mast cells in various tissues and organs (1). The spectrum of this condition ranges from cutaneous mastocytosis (CM) to aggressive systemic mastocytosis (SM) (1). Compared with SM, CM is regarded as a benign variant confined to the skin, with indolent progression and a favorable prognosis (2). However, our previous study found that Chinese patients tend to have persistent lesions without a tendency to regress, leading to relatively poor long-term outcomes (3). Accordingly, key knowledge gaps remain regarding the detailed pathogenic factors in Chinese patients, as well as their potential correlation with clinical phenotype and disease course.

Histopathological analysis allows the characterization of mast cell infiltration patterns and the detection of core immunophenotypic markers such as CD117, CD25, and tryptase (2), and thus plays an important role in the diagnosis of mastocytosis. Despite the advances in diagnostic criteria introduced by the 2022 World Health Organization (WHO) classification (4), pathological features and disease-related parameters remain poorly defined in Chinese patients with different CM subtypes (5, 6). Therefore, this study provides a comprehensive analysis of the pathological morphology and immunophenotype among CM subtypes and explores the potential association between mast cell-related pathogenic features and clinical phenotypes.

## Methods

### Patients

**Figure 1:**
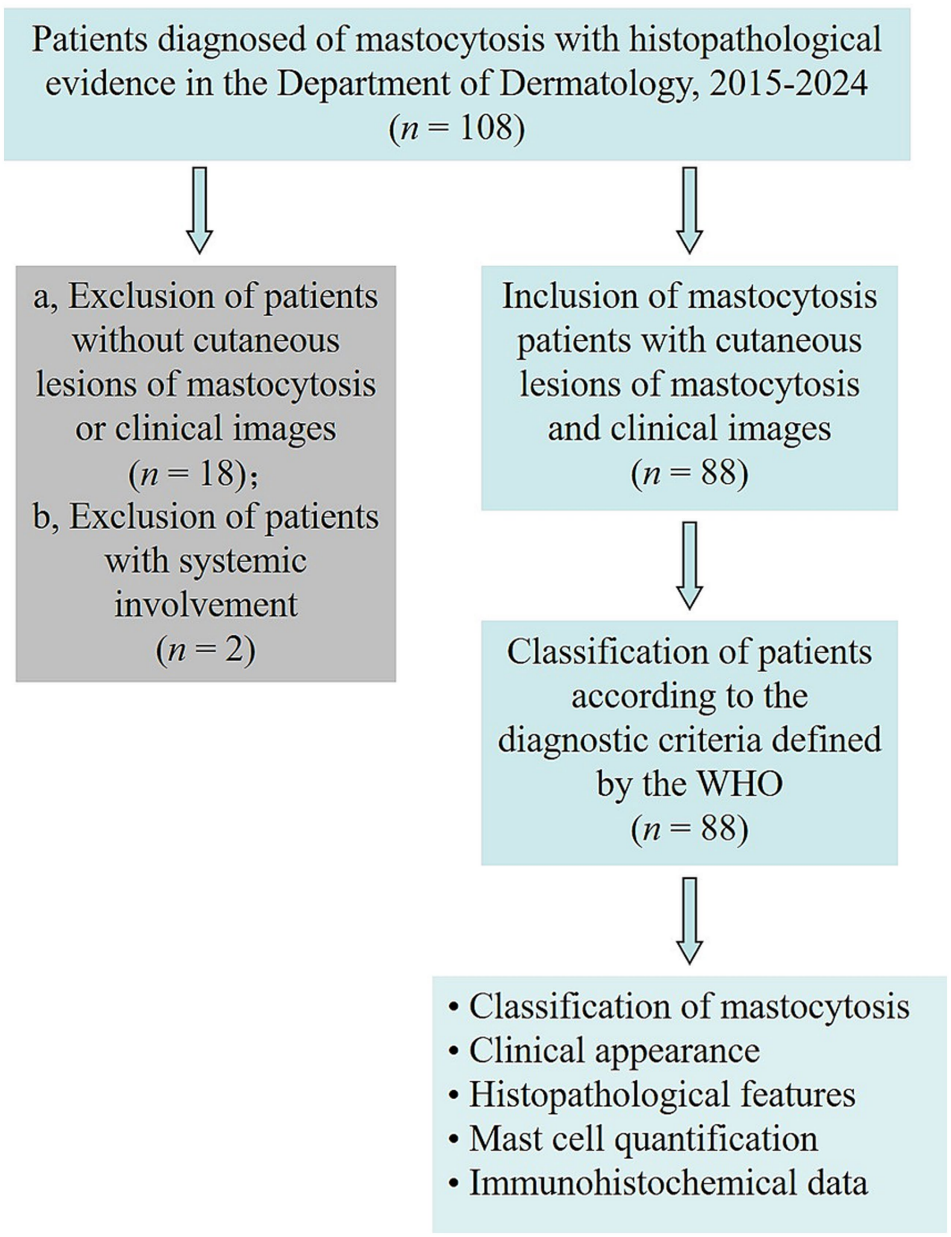
The flowchart of this study.

Our study reviewed all patients diagnosed with CM in the Department of Dermatology, Peking Union Medical College Hospital, Beijing, China, between 2015 and 2024 (Figure 1). The inclusion criterion was the presence of cutaneous lesions consistent with mastocytosis, confirmed by histopathological examination, in accordance with the WHO criteria for CM. Patients without histological confirmation, those with systemic involvement, or those with concurrent hematological malignancies were excluded. Clinical images and histopathological data were reviewed by experienced dermatologists and pathologists. Written informed consent for clinical images, biopsy, and histopathological analysis was obtained from all patients. The study was conducted in accordance with the Declaration of Helsinki, and ethical approval was obtained from the Institutional Review Board of Peking Union Medical College Hospital.

### Classification and diagnostic criteria

All included patients were classified into different categories according to the diagnostic criteria defined by the 2022 WHO classification, including maculopapular CM (MPCM), diffuse CM (DCM), and cutaneous mastocytoma (4). MPCM is characterized by small reddish or pigmented macules and papules. MPCM can be classified into a pigmented variant (also known as urticaria pigmentosa, UP) and a non-pigmented variant, which was formerly called telangiectasia macularis eruptive perstans (TMEP), and is defined by the presence of typical telangiectatic macules (7). DCM usually shows a widespread and diffuse pattern with a thickened, yellowish-brown appearance. Cutaneous mastocytoma presents as a solitary lesion or a few localized nodules or plaques with diameters of 1–5 cm and typically occurs within a few months after birth.

### Histopathological assessment and mast cell burden scoring

Immunohistochemical analysis was performed using antibodies against mast cell tryptase, CD117, CD2, CD25, and CD30. An antibody against tryptase was used as a standard marker to quantify mast cells and evaluate the degree of mast cell infiltration. Two experienced dermatopathologists independently evaluated all slides in a blinded manner. Three representative, non-overlapping high-power fields (HPFs, ×400 magnification) with the densest mast cell infiltration were selected in each specimen, and the average number of mast cells per HPF was calculated. A semi-quantitative scoring system was used to evaluate mast cell burden on tryptase- and CD117-stained slides:

Score 1: <20 mast cells/HPF, sparse scattered distribution;

Score 2: <50 mast cells/HPF, mildly increased density with occasional small aggregates;

Score 3: <100 mast cells/HPF, moderately dense infiltration with small clusters;

Score 4: <250 mast cells/HPF, dense infiltration with large confluent aggregates;

Score 5: ≥250 mast cells/HPF, massive sheet-like dense infiltration.

The presence of specific membrane/cytoplasmic/intracellular immunoreactivity was considered positive for CD2, CD25, and CD30. Positive controls included lymph node tissue for CD117 and CD30, and tonsil tissue for tryptase, CD2, and CD25. Negative controls consisted of non-lesional skin and omission of the primary antibody. Interobserver agreement was assessed in 20% of randomly selected cases (*n* = 18) using the weighted kappa coefficient, revealing substantial agreement (*κ* = 0.82, *p* < 0.001).

### Data collection and analysis

The following data were collected: age, sex, mastocytosis subtype, clinical appearance, pathological features, degree of mast cell infiltration, and immunohistochemical findings. The follow-up evaluation was performed via phone at the end of this study.

Statistical analysis was performed using SPSS statistical software (version 28.0) with the Kruskal–Wallis test for the ordinal variables, and Mann–Whitney U and Fisher’s exact tests for categorical variables. The Bonferroni correction was applied to multiple comparisons, with effect sizes calculated to quantify the strength of associations. Multivariate logistic regression analyses were performed for categorical variables to control for potential confounding variables, including age and sex. Statistical significance was set at *p* < 0.05.

## Results

### Comparison of mast cell burden among different CM subtypes

The study enrolled 88 CM patients, including 49 adult-onset patients (>16 years old, 55.68%) and 39 pediatric-onset patients (≤16 years old, 44.32%). The most common subtype was UP (70, 79.55%), followed by TMEP (11, 12.50%), cutaneous mastocytoma (5, 5.68%), and DCM (2, 2.27%; Table 1). Among the 39 pediatric patients, the distribution of CM subtypes was as follows: UP in 32 patients (82.05%), DCM in 3 patients (7.69%), and mastocytoma in 4 patients (10.26%). The male-to-female ratio was 2.55:1. In pediatric patients with UP, lesions were predominantly distributed on the trunk and extremities, with facial involvement observed in 12 patients (37.50%), neck involvement in 10 (31.25%), and scalp involvement in 5 (15.63%). In DCM, lesions showed diffuse involvement of the scalp, face, neck, trunk, and extremities. In mastocytoma, solitary lesions were located on a finger, the buttock, the rib area, and the back, respectively.

**Table 1:** The histopathological and immunohistochemical features of cutaneous mastocytosis.

Parameters	All	UP	TMEP	DCM	Mastocytoma
All	88	70	11	2	5
Mast cell infiltrate scores					
1	27	18	9	0	0
2	30	27	2	0	1
3	13	13	0	0	0
4	4	3	0	0	1
5	14	9	0	2	3
Pathological features					
Atrophic epidermis	15	11	4	0	0
Acanthotic epidermis	7	5	1	0	1
Increased basal layer Pigmentation	62	54	8	0	0
Dilated blood vessels	8	4	4	0	0
Inflammatory cells					
Lymphocytes and histiocytes	62	50	10	0	2
Eosinophils	11	10	0	0	1
Neutrophils	1	1	0	0	0
Inflammation pattern					
Perivascular infiltrate	54	44	10	0	0
Dense or ribbon infiltrate	8	5	0	0	3
Immunophenotypes					
CD117	88	70	11	2	5
CD2	42	35	0	2	5
CD25	15	13	0	0	2
CD30	12	9	0	0	3
					

	Clinical features	Pathological features	Potential implication
Clinical subtype and mast cell burden	UP	Moderate mast cell burden in the dermis	Mast cell burden: TMEP < UP < DCM/mastocytoma
	TMEP	Low burden with loosely scattered mast cells	
	DCM/mastocytoma	Markedly higher mast cell burden	
Clinical subtype and CD2 expression	TMEP	CD2(−)	CD2 expression potentially associated with high levels of mast cell burden
	DCM/mastocytoma/half UP	CD2(+)	
Congenital lesion and CD25/30 expression	Most congenital lesions	CD25(+)/CD30(+)	CD25/30 expression potentially associated with specific pediatric subtypes with high levels of mast cell burden
	Other	CD25(−), CD30(−)	
UP morphology and mast cell burden	Lesion size	Mast cell burden	Small < variable/large lesion Confounded by age (younger age associated with higher mast cell burden)
	Elevated versus flat lesion	Mast cell burden	Flat < elevated lesion

UP, urticaria pigmentosa; TMEP, telangiectasia macularis eruptive perstans; DCM, diffuse cutaneous mastocytosis.

**Figure 2:**
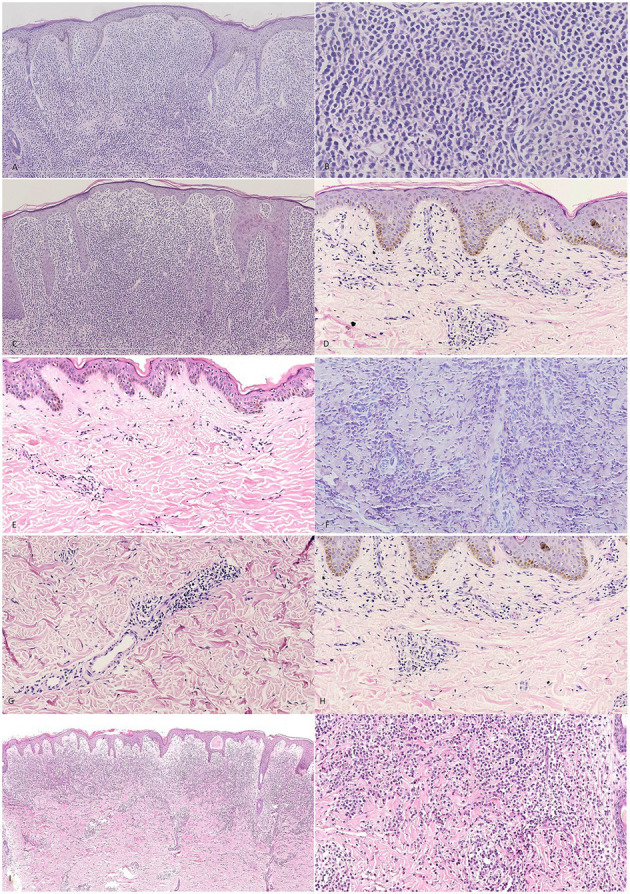
Histopathological features of CM subtypes. **(A)** Histopathologic examination of DCM lesions revealed diffuse infiltration and accumulation of abundant mast cells throughout the dermis. **(B)** Mast cells are round or oval, with abundant cytoplasm and numerous basophilic granules. The nuclei are round or reniform with fine chromatin and indistinct nucleoli. **(C)** A skin specimen of cutaneous mastocytoma showed a densely infiltrating neoplasm of numerous mast cells. **(D)** UP presented with moderate infiltration of mast cells with sheet-like and nested patterns in the dermis. **(E)** TMEP exhibited loosely scattered mast cells at the periphery of dilated blood vessels in the upper dermis. **(F)** Basophilic granules in mast cells stained positive for toluidine blue. **(G)** Dilated blood vessels were present in the dermis of UP and TMEP. **(H)** The majority of cases showed sparse to moderate inflammatory infiltrates, predominantly lymphocytes and histiocytes. **(I)** A dense or ribbon-like inflammation pattern was also seen in the dermis of several UP cases. **(J)** Dermal infiltration with eosinophils, neutrophils, and nuclear dust was seen in one UP case. (**A–E,G–J**, H&E stain; **F**, toluidine blue stain). UP, urticaria pigmentosa; TMEP, telangiectasia macularis eruptive perstans; DCM, diffuse cutaneous mastocytosis.

UP was the most common subtype in both adult- and pediatric-onset groups, accounting for 77.55% (38/49) of adults and 82.05% (32/39) of children, with no significant difference in proportion between the two groups. All CM cases revealed monomorphic mast cell infiltrates of various degrees. Patients with DCM presented with diffuse infiltration of mast cells throughout the dermis (Figures 2A,B). Patients with mastocytoma had a localized dermal neoplasm consisting of abundant mast cells (Figure 2C). By contrast, UP (Figure 2D) and TMEP (Figure 2E) showed a relatively lower mast cell burden, especially TMEP, which had merely increased mast cells at the periphery of blood vessels in the upper dermis. Mast cells in CM are round or oval, with abundant cytoplasm and numerous basophilic granules as demonstrated by toluidine blue staining (Figure 2F).

To investigate whether mast cell burden may serve as a distinguishing feature of different subtypes, the degree of mast cell infiltration was analyzed. Overall, 27 cases (30.68%) had mast cell infiltration scores of 1, 30 cases (34.09%) had scores of 2, 13 cases (14.77%) had scores of 3, four cases (4.55%) had scores of 4, and 14 cases (15.91%) had scores of 5 (Table 1). The mean mast cell score was 2.41 in total, 2.40 for UP, 1.18 for TMEP, 5.00 for DCM, and 4.20 for mastocytoma. Among UP cases, those with scores of 1–3 accounted for 82.86% (58/70). Of the 11 TMEP cases, 9 (81.82%) scored 1, with the remaining 2 scoring 2. All DCM cases were graded 5. Mast cell aggregation was markedly dense in DCM and mastocytoma. Mastocytoma scores varied from 2 to 5, with 3 cases (60.00%) scoring 5. Significant differences in mast cell burden were observed among subtypes (*p* < 0.001), with an overall effect size of ε^2^ = 0.23, indicating a moderate effect. Multivariate logistic regression analysis, adjusted for age and sex, confirmed overall differences among subtypes (*p* < 0.001). Using UP as the reference, TMEP showed lower mast cell burden [adjusted odds ratio (OR) = 0.12, 95% confidence interval (CI): 0.02–0.68, *p* = 0.016], whereas mastocytoma was associated with higher infiltration (adjusted OR = 7.73, 95% CI: 1.24–48.15, *p* = 0.028) (Table 1).

### Other pathological features of CM subtypes

Epidermal changes were observed in 78 (88.64%) patients and were categorized into three main patterns: normal, acanthotic, or atrophic. Normal epidermis was observed in the majority of cases (66, 75.00%). Acanthosis was seen in 7 cases (7.95%), mostly in the UP subgroup. Atrophic epidermis was observed in 15 cases (17.05%). Notably, atrophic change was more frequent in TMEP (4/11, 36.36%) compared with UP (11/70, 15.71%) (*p* > 0.05). The majority of cases in the UP and TMEP groups presented increased pigmentation in the basal layer (77.14 and 72.73%, respectively). Liquefaction degeneration of basal cells was restricted to four UP cases.

Dilated blood vessels were observed in 4/70 (5.71%) of UP cases and 4/11 (36.36%) of TMEP cases, and the proportion was significantly higher in TMEP (*p* < 0.05). In all cases with vascular ectasia, mast cell infiltration was consistently observed at the periphery of the dilated vessels (Figure 2G). Regarding inflammation, the majority of cases (71.59%) showed inflammatory infiltrates, predominantly lymphocytes and histiocytes (98.41%) (Figures 2H,I). Eosinophils were noted in 11 cases (12.50%), and neutrophils and nuclear dust were seen in one case with UP (Figure 2J). The incidence of eosinophils was 14.29% in the UP group but not in the TMEP group. Regarding the inflammation pattern, the majority of UP cases exhibited moderate inflammatory infiltrates, whereas five cases (7.14%) displayed a dense or ribbon-like infiltrate pattern (Figure 2I). Almost all TMEP cases (90.91%) showed sparse perivascular inflammation (Figure 2E). Interestingly, minimal dermal changes were observed, especially in collagen fibers. A thickened dermis and collagen fiber hyperplasia were present in three UP cases and one TMEP case. Mucoid degeneration of fibroblasts was noted in the dermis of one TMEP case. Hyaline degeneration was observed in the superficial dermis of one UP case, demonstrating the absence of elastic fibers on elastic tissue staining. No atypical pleomorphic cells, mitotic figures, necrosis, or ulceration were observed in this study.

### Comparison of immunophenotypes among CM subtypes

To investigate whether different CM subtypes exhibit distinct immunophenotypes, immunohistochemical analysis was performed for mast cell tryptase, CD117, CD2, CD25, and CD30.

**Figure 3:**
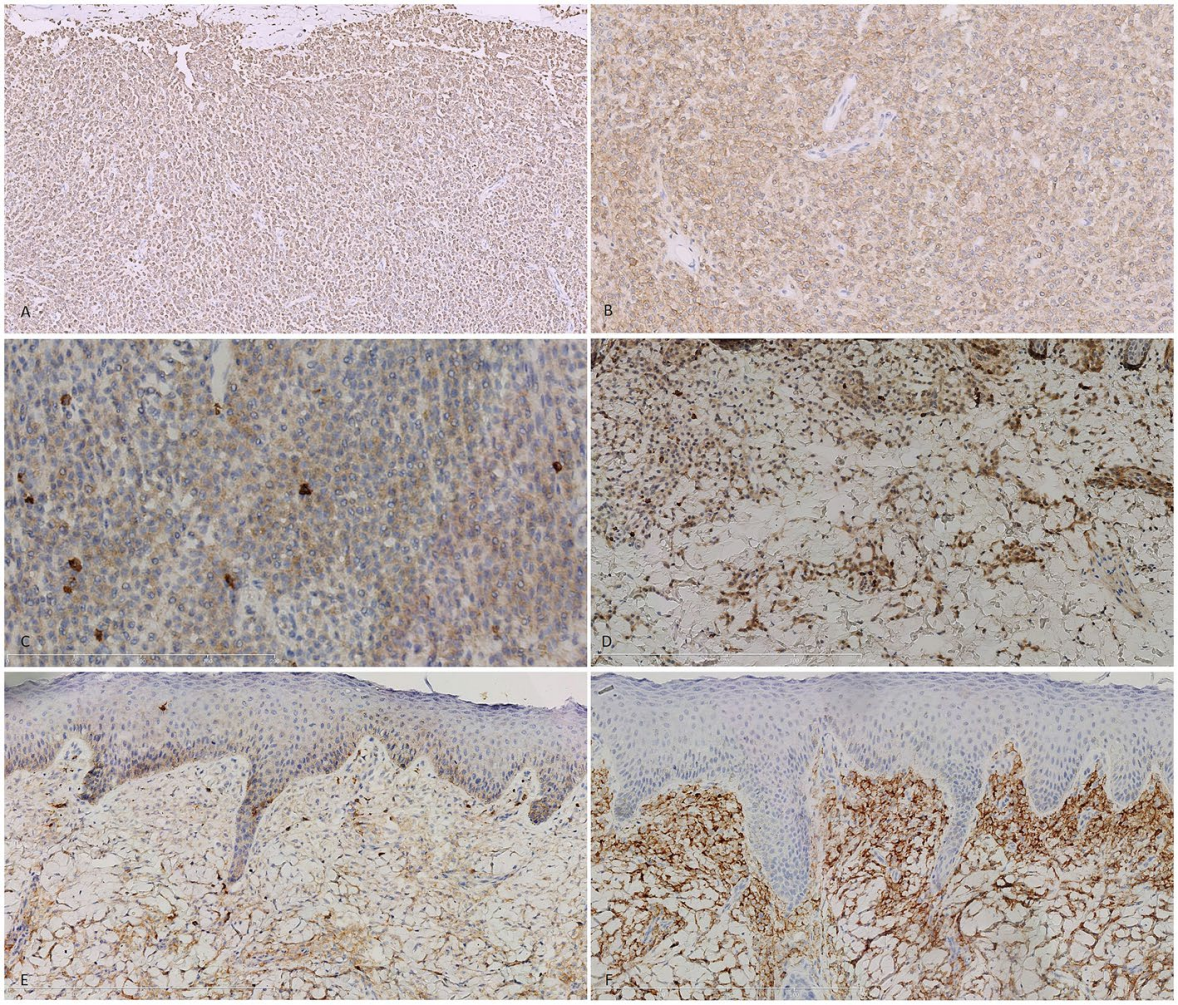
Immunohistochemical features of CM. Immunostaining of mast cell tryptase **(A)** and CD117 **(B)** showed diffuse infiltration of abundant mast cells in the dermis. CD2 expression was observed in numerous mast cells infiltrating the dermis of the mastocytoma **(C)**. Many UP cases clearly showed CD2-positive immunophenotypes **(D)**. Several cases of congenital nodular mastocytoma exhibited positivity for CD25 **(E)** and CD30 **(F)**. (S-P stain). UP, urticaria pigmentosa; CM, cutaneous mastocytosis.

CD117 was expressed in all cases, with staining scores comparable to those of tryptase in mast cell infiltrates. CD2 was positive in all patients with DCM and mastocytoma, as well as in 35 patients with UP, but was negative in TMEP. CD2 expression differed significantly between TMEP and other subtypes (*p* < 0.001, effect size of Cramér’s *V* = 0.394). Using non-TMEP as the reference, Firth penalized logistic regression showed that TMEP was associated with lower CD2 positivity (adjusted OR = 0.042, 95% CI: 0.002–0.850, *p* = 0.039) (Table 1). The proportion of CD2-positive mast cells varied from patient to patient, ranging from 20 to 100%. CD25 was expressed in 17.05% (15/88) of the cases, mainly in congenital mastocytoma nodules and UP. CD30 expression was detected in 13.64% (12/88) of the cases, including three congenital mastocytoma cases and seven congenital UP nodules. Compared with CD2, the expression of CD25 and CD30 was lower and might correlate with congenital nodules. In summary, UP was positive for CD117 and showed variable positivity for CD2, CD25, and CD30, whereas TMEP was positive only for CD117. Both mastocytoma and DCM were positive for CD117 and CD2, with mastocytoma additionally showing partial positivity for CD25 and CD30. Representative immunostained images are shown in Figure 3, and the results are summarized in Table 1.

### Correlation between morphology of cutaneous lesions and pathological parameters in UP

Cutaneous lesions of UP display heterogeneous morphology and arrangement. To explore the association between pathological parameters and clinical manifestations, UP lesions were categorized as monomorphic or polymorphic in shape; small (<1 cm in diameter), large (>1 cm in diameter), or variable in size; flat or elevated; and confluent or non-confluent (Supplementary Figure 1).

As shown in Supplementary Table 1, 41 patients had monomorphic lesions and 29 patients had polymorphic lesions. The mean scores for mast cell infiltration were 2.10 and 2.76, respectively. Epidermal intracellular edema was observed in 4.88 and 24.14% of the monomorphic and polymorphic groups, respectively. Among the cases with dense dermal inflammation, 80.00% showed polymorphic lesions, while only one case displayed monomorphic lesions.

Regarding lesion size, 41 cases (58.57%) had small lesions, 21 (30.00%) had large lesions, and 8 (11.43%) had variable sizes. The median mast cell burden was 2.0 in the small group and 2.5 in the non-small group (*p* < 0.05, rank-biserial *r* = −0.266, 95% CI: −0.535 to 0.004). Multivariate logistic regression adjusted for sex and age revealed that lesion size was no longer significantly associated with mast cell burden. By contrast, age remained independently associated (adjusted OR = 0.96, 95% CI: 0.93–0.99, *p* = 0.012), with younger age associated with higher mast cell burden. Over half of the large lesions scored 3–5, including 33.33% (7/21) with a score of 5, whereas the majority of small lesions (75.61%) scored ≤2. Among cases with epidermal intracellular edema, 77.78% had large or variable lesions, while only two cases displayed small lesions. All patients with dense dermal inflammation exhibited large lesions.

Flat lesions were observed in 53 patients (75.71%), and elevated lesions in 17 patients (24.29%). The mean mast cell scores were 1.91 and 3.65, respectively (*p* < 0.001, rank-biserial correlation = −0.58, 95% CI: −0.77 to −0.37), indicating a large effect. Using flat lesions as the reference, multivariate logistic regression analysis adjusted for age and sex revealed that elevated lesions were associated with higher mast cell burden (adjusted OR = 11.4, 95% CI: 3.97–32.75, *p* < 0.001) (Table 1). The majority of flat lesions (77.36%) scored 1 or 2, while 70.59% of elevated lesions scored >2, including eight cases with a score of 5. Among the 16 cases with stratum spinosum thickness changes, 75.00% displayed elevated lesions, while only 4 cases had flat lesions. All elevated cases showed increased basal layer pigmentation, whereas 30.19% of the flat lesions showed no obvious changes. All patients with dense dermal inflammation exhibited elevated lesions. In addition, 12 cases (17.14%) showed confluent lesions, and the remaining 58 showed no signs of confluence.

### Clinical outcomes in Chinese patients with CM

A total of 59 patients underwent follow-up evaluation. The follow-up duration ranged from 1 to 10 years. The mean disease duration from the initial onset of skin lesions to complete remission or the study endpoint was 6.10 ± 6.93 years (range, 0.75–29.50 years), and 29 patients had disease persistence exceeding 8 years without complete remission to date. There were 4 (6.78%) patients with complete remission, 20 (33.90%) patients with partial remission (characterized by reduced lesion size, lighter color and lower elevation, and decreased symptom frequency), 24 (40.68%) patients with non-remission, and 9 (15.25%) patients with disease progression. These findings suggest that CM lesions tend to be persistent in Chinese patients. No significant differences in patient outcomes were observed between different variants or subtypes with different lesion morphology (*p* > 0.05).

## Discussion

This study provides a detailed description of the histopathological features and immunophenotypes of CM. Analysis of histopathological characteristics revealed a significant association between mast cell burden and CM subtypes. Patients with UP showed moderate mast cell infiltration in the dermis. However, TMEP lesions exhibited mild infiltration of loosely scattered mast cells, sometimes with an overlap between mastocytosis and normal skin. In contrast, the DCM and mastocytoma groups showed markedly higher levels of mast cell burden. These findings indicate that mast cell burden may serve as a potential distinguishing feature of different CM subtypes.

In addition, our study found that UP and TMEP have several other pathological differences. UP usually shows a normal or acanthotic epidermis, whereas TMEP tends to have a normal or atrophic epidermis. In contrast to TMEP, UP has several unique pathological features, including parakeratosis, focal intracellular edema, parakeratotic cells, mild acanthosis, focal liquefaction degeneration of basal cells (these changes were mild and did not constitute a spongiotic or psoriasiform pattern), eosinophil and neutrophil infiltration, and a dense inflammatory pattern. Although the difference in eosinophil infiltration between UP and TMEP did not reach statistical significance (*p* > 0.05, likely due to the small sample size of TMEP), the complete absence of eosinophils in TMEP may be a helpful negative diagnostic clue. In addition, a dense or ribbon-like pattern, when present, is highly suggestive of UP rather than TMEP. Furthermore, TMEP demonstrates an atrophic epidermal pattern more frequently than UP. The higher proportion of dilated vessels in TMEP suggests that while vascular ectasia is not absolutely specific for TMEP, its presence, accompanied by perivascular mast cell infiltration, may raise suspicion for TMEP. Interestingly, the majority of cases in both UP and TMEP groups presented with increased basal layer pigmentation. Regarding the classification of MPCM, some authors described UP as a pigmented variant and TMEP as a non-pigmented variant (7), which seems inaccurate given the lack of supporting pathological evidence.

Moreover, the serine protease tryptase is strongly expressed by all mast cells and is considered the most reliable marker for the identification of atypical and hypogranulated cells, even with very small infiltrates (8). Our results demonstrated that CD117 had high sensitivity, similar to tryptase in mastocytosis, and could also be a helpful marker for the detection of mast cells. We also discovered that different CM subtypes exhibit various immunophenotypes. UP was positive for CD117 and partially positive for CD2, CD25, and CD30, whereas TMEP was positive only for CD117. Both mastocytoma and DCM were positive for CD117 and CD2, whereas mastocytoma showed partial positivity for CD25 and CD30. Compared with UP, TMEP showed significant differences in the expression pattern of CD2, CD25, and CD30.

CD2, CD25. and CD30 are aberrant markers of neoplastic mast cells, which are included in WHO diagnostic criteria for SM (4). Expression of CD2, CD25, and/or CD30 is demonstrable in the majority of patients with SM (9–11). By contrast, normal mast cells usually lack CD2, CD25, and CD30 (12, 13). Notably, neoplastic mast cells in CM tend to have normal cytological features and do not differ from reactive mast cells.6 However, several studies have shown that CD25 expression in mast cells in the skin is predictive of SM (14, 15). In this study, CD2 was expressed in almost half of the cases analyzed, including all cases with DCM and mastocytoma, and half of the UP cases. CD2 expression differed between TMEP and other subtypes, suggesting that CD2 expression may be associated with high levels of mast cell infiltration. Our results indicate that CD2 expression is not specific to malignant mastocytosis but is also observed in patients with CM. Interestingly, CD25 was expressed in some congenital nodules of UP and mastocytoma, while CD30 was detected in several congenital nodules in our series. Therefore, CD25 and CD30 may potentially correlate with specific pediatric subtypes with high levels of mast cell infiltration. The immunophenotypic profile of CM and the specific marker for neoplastic mast cells in the skin still require further elucidation.

The factors associated with variable morphologies remain largely unknown. Brockow et al. (16) reported that MPCM lesions in children are larger than those in adults. Similarly, Wiechers et al. (17) observed that pediatric patients with large MPCM lesions had significantly lower tryptase levels, earlier disease onset, and shorter disease duration than those with small lesions. This study explored the association between distinct UP morphological features and pathological parameters. Although larger lesions appeared to correlate with higher mast cell burden, we found that this association was confounded by age, and that younger age was associated with higher mast cell burden. Our findings indicate that larger lesions may be related to earlier disease onset, which is consistent with the above literature and our previous report (3). Accordingly, age appears to be the primary confounding factor driving the original association between lesion size and mast cell burden. Earlier onset age might be correlated with the expansion and accumulation of mast cells during disease progression. In addition, elevated lesions showed much larger numbers of mast cells than flat lesions. Our results demonstrated that elevated lesions were potentially associated with a high degree of mast cell infiltration.

This study has several limitations. First, it is a retrospective, single-center study with potential selection bias and an inability to fully control for confounding variables. Second, small sample sizes in the DCM and mastocytoma subtypes restrict the statistical power of subgroup analyses. Third, the study lacked molecular data, including KIT mutations. Due to the mild nature of CM and the absence of systemic involvement, the majority of patients would not agree to undergo genetic analysis. Other limitations include the lack of standardized morphological classification criteria, a semi-quantitative mast cell scoring system, and limited external validity due to the ethnic and geographic constraints of the single-center cohort.

In conclusion, mast cell burden is associated with CM subtypes, and higher mast cell burden may correlate with specific subtypes, clinical morphologies, and earlier disease onset. CD2, CD25, and CD30 might be correlated with specific subtypes with high mast cell burden and have potential value in CM diagnosis and subtyping. Given significant pathological differences, TMEP may represent a unique and rare morphological variant of UP. Larger prospective and molecular studies are warranted to confirm these findings.

## Data Availability

The raw data supporting the conclusions of this article will be made available by the authors, without undue reservation.
