# Supplementary figures and images for: Mast cell burden and immunophenotype of Chinese patients with cutaneous mastocytosis: a 10-year study with focus on the easily neglected pathogenic features
Source: Front Med (Lausanne). 2026 Jun 22;13:1828727. doi: 10.3389/fmed.2026.1828727 (PMC13335676; doi:10.3389/fmed.2026.1828727)

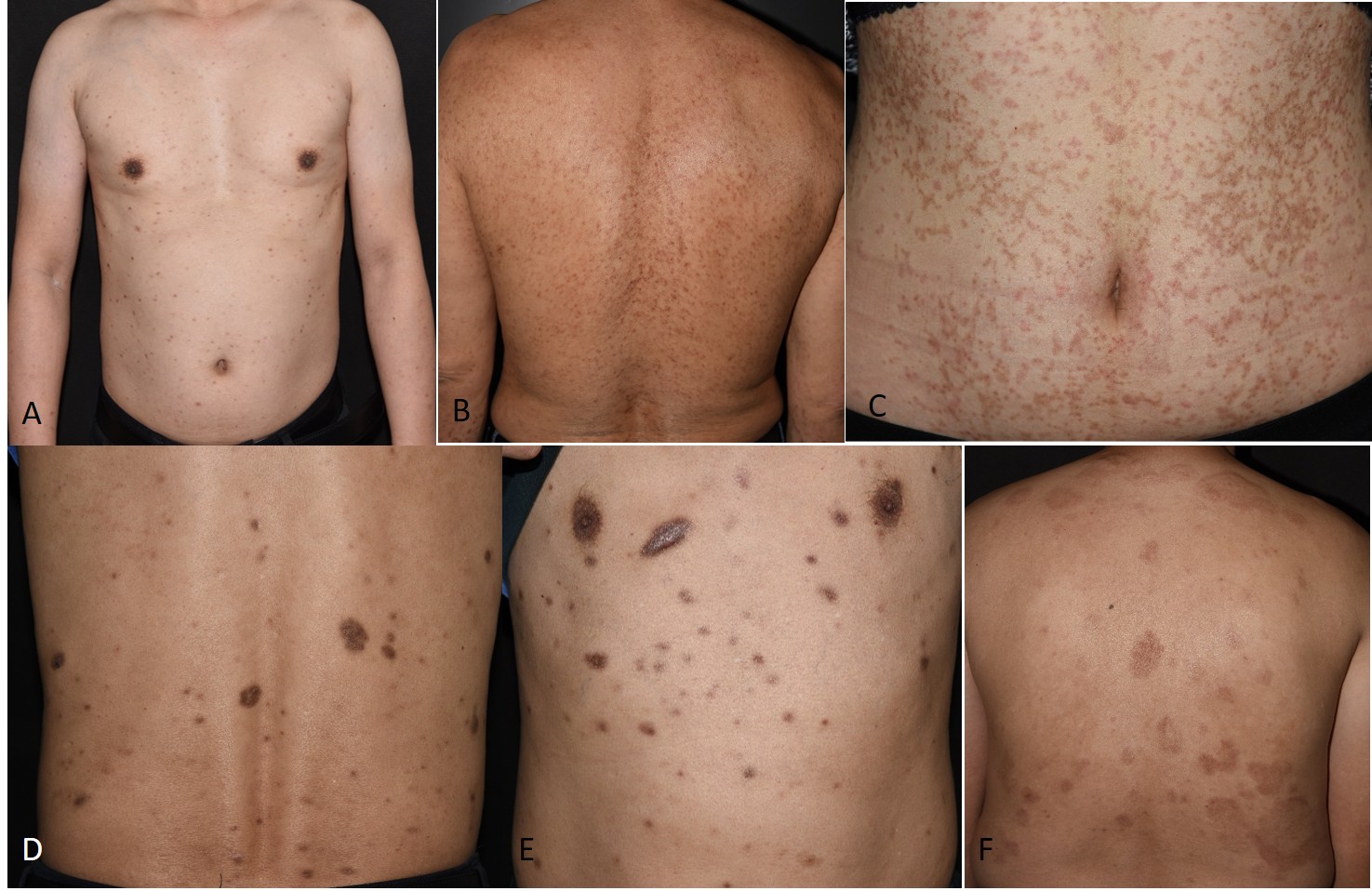

Supplement: Supplementary file 1 [file Image_1.jpeg]
