# Supplementary material for: Mast cell burden and immunophenotype of Chinese patients with cutaneous mastocytosis: a 10-year study with focus on the easily neglected pathogenic features
Source: Front Med (Lausanne). 2026 Jun 22;13:1828727. doi: 10.3389/fmed.2026.1828727 (PMC13335676; doi:10.3389/fmed.2026.1828727)
Supplement: Supplementary file 2 [file Supplementary_File_1.doc]

**Supplementary Table 1**. The histopathological features in UP cases with different morphology

| Parameters | Shape | | Size | | | Elevation | | | Arrangement | | |
| --- | --- | --- | --- | --- | --- | --- | --- | --- | --- | --- | --- |
|  | Monomorphic | Polymorphic | Small | Large | Variable | | Flat | Elevated | | Confluent | Non-confluent |
| All | 41 | 29 | 41 | 21 | 8 | | 53 | 17 | | 12 | 58 |
| Scores of mast cell infiltrate | | | | | | | | | | | |
| 1 | 13 | 6 | 12 | 4 | 2 | | 22 | 0 | | 2 | 16 |
| 2 | 17 | 10 | 19 | 6 | 2 | | 19 | 5 | | 5 | 22 |
| 3 | 8 | 4 | 8 | 3 | 2 | | 8 | 4 | | 3 | 10 |
| 4 | 0 | 3 | 0 | 1 | 2 | | 3 | 0 | | 2 | 1 |
| 5 | 3 | 6 | 2 | 7 | 0 | | 1 | 8 | | 0 | 9 |
| Pathological features | | | | | | | | | | | |
| Atrophic epidermis | 6 | 5 | 6 | 3 | 2 | | 3 | 8 | | 3 | 8 |
| Acanthotic epidermis | 3 | 2 | 3 | 1 | 1 | | 1 | 4 | | 2 | 3 |
| Increased pigmentation | 32 | 22 | 32 | 16 | 6 | | 33 | 21 | | 10 | 44 |
| Dilated blood vessels | 3 | 1 | 3 | 1 | 0 | | 2 | 2 | | 2 | 2 |
| Inflammatory cells | | | | | | | | | | | |
| Eosinophils | 6 | 4 | 6 | 4 | 0 | | 6 | 4 | | 1 | 9 |
| Neutrophils | 0 | 1 | 0 | 1 | 0 | | 0 | 1 | | 0 | 1 |
| Inflammation pattern | | | | | | | | | | | |
| perivascular infiltrate | 27 | 17 | 30 | 10 | 4 | | 27 | 17 | | 10 | 34 |
| Dense or ribbon infiltrate | 1 | 4 | 0 | 5 | 0 | | 0 | 5 | | 0 | 5 |

**Supplementary Figure 1**.

UP were defined as monomorphic (A-C) and polymorphic (D) in shape; small (A-C), variable (D-E) and large (F) in size; flat (A-D) and elevated (E, F); and confluent (C, F).
